# Economy and Hospital Gardens

**Published:** 1915-07-31

**Authors:** 


*To the Editor of* The Hospital.

### Economy and Hospital Gardens.

*To the Editor of* The Hospital.

Sir,—At this time, when national and individual economy is everywhere being preached, institutional economy is something that cannot be lost sight of. When on a holiday at Worthing I was very much struck by the use to which the local hospital put its spare ground. True, there were a few flower beds visible, but nearly every available space was being utilised for the cultivation of potatoes and other vegetable crops presumably for the use of the institution.

Might not other hospitals follow this example?—Yours faithfully, Economy.

